# Supplementary figures and images for: EBV+ tumors exploit tumor cell-intrinsic and -extrinsic mechanisms to produce regulatory T cell-recruiting chemokines CCL17 and CCL22
Source: PLoS Pathog. 2022 Jan 13;18(1):e1010200. doi: 10.1371/journal.ppat.1010200 (PMC8791514; doi:10.1371/journal.ppat.1010200)

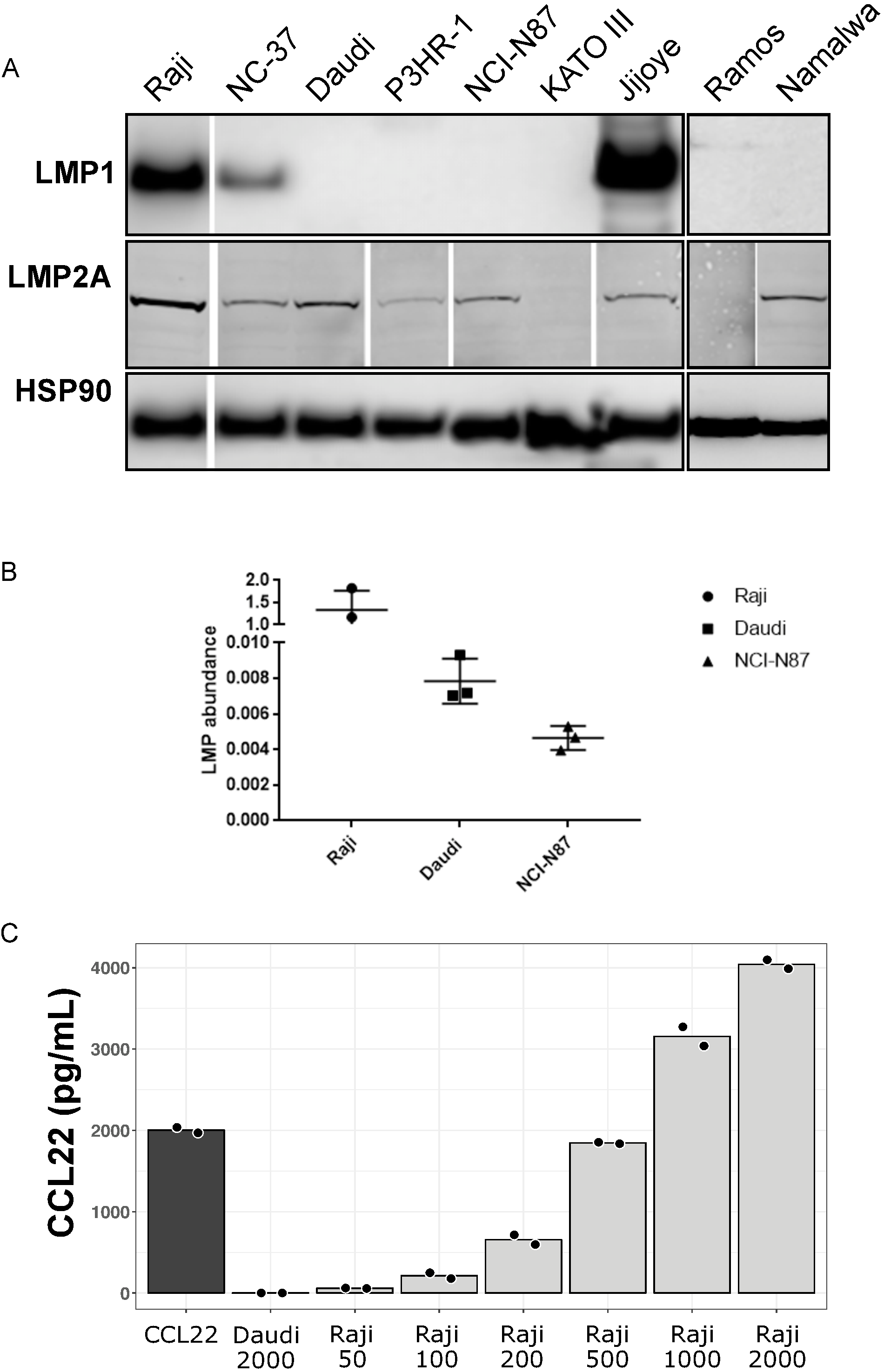

Supplement: S1 Fig — (A) Western blots on 50 μg of protein lysate from 9 human cell lines were probed for LMP1, LMP2A, and HSP90 as indicated. Ramos and Namalwa were run on a separate gel. (B) RT-PCR for LMP1/2 in three cell lines confirms detection of LMP transcript in NCI-N87. (C) Supernatants from increasing numbers of seeded Raji cells (50 to 2000 thousand cells per well) or from 2000 Daudi cells grown for 24 hours, or a standard of 2000 pg/mL CCL22 quantitated by CCL22 ELISA. Where bands from the same Western blot have been reordered to match across analytes, white gaps are shown–LMP1 and HSP90 were probed on a separate blot from LMP2A. Ramos and Namalwa lysates were run on a separate gel, visually separated by a black border, from the other lysates. (TIF) [file ppat.1010200.s001.tif]

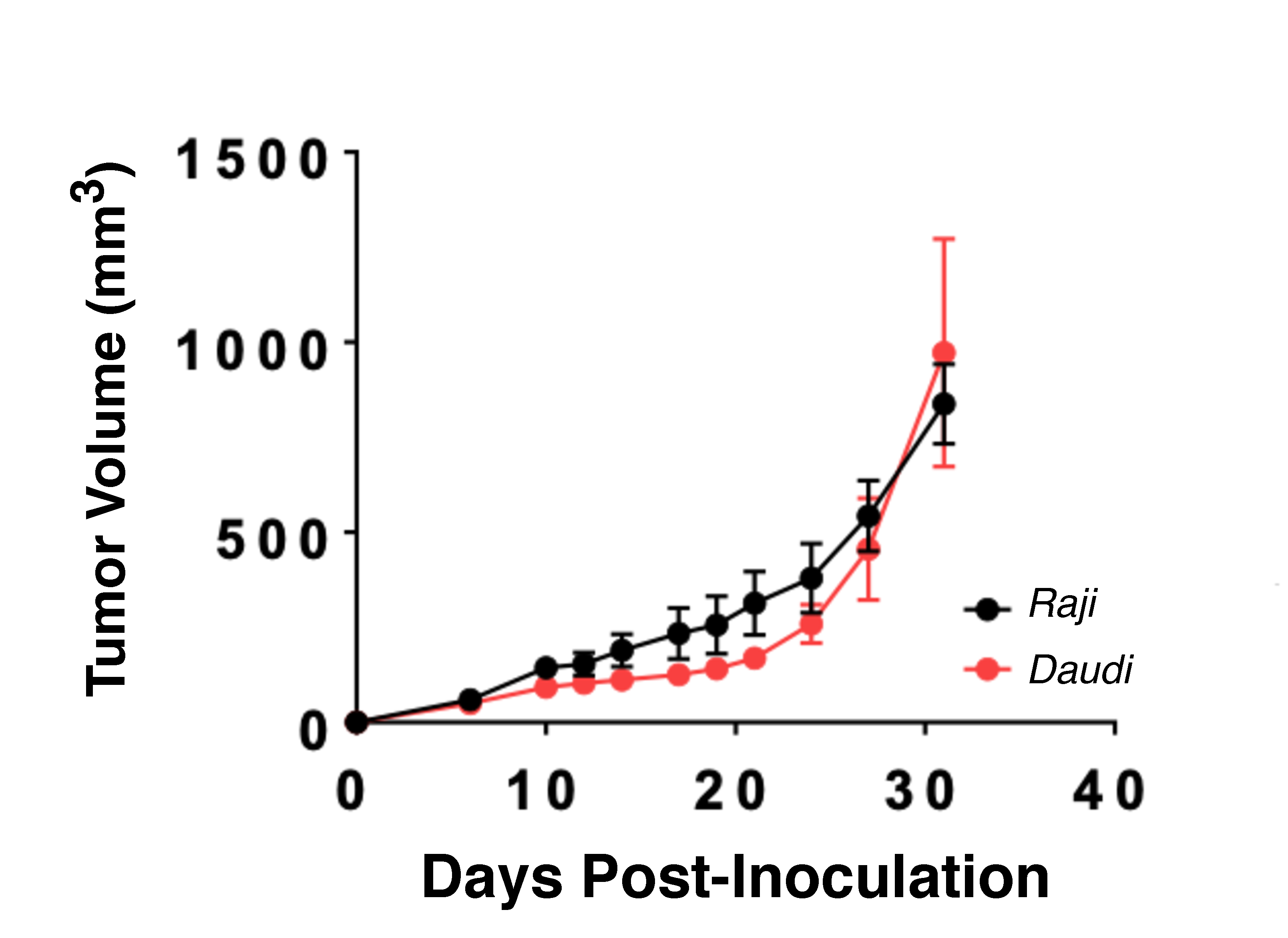

Supplement: S2 Fig — Raji and Daudi Xenografts were established in NOD/SCID mice to measure chemokine production and iTreg migration (Fig 2C and 2D). Tumor size was measured by calipers regularly over 30 days post- inoculation with 2 x 10^6^ of Raji or Daudi cells, as indicated. Curves show the means and standard deviations for calculated tumor volumes from 5 mice per tumor type. (TIF) [file ppat.1010200.s002.tif]

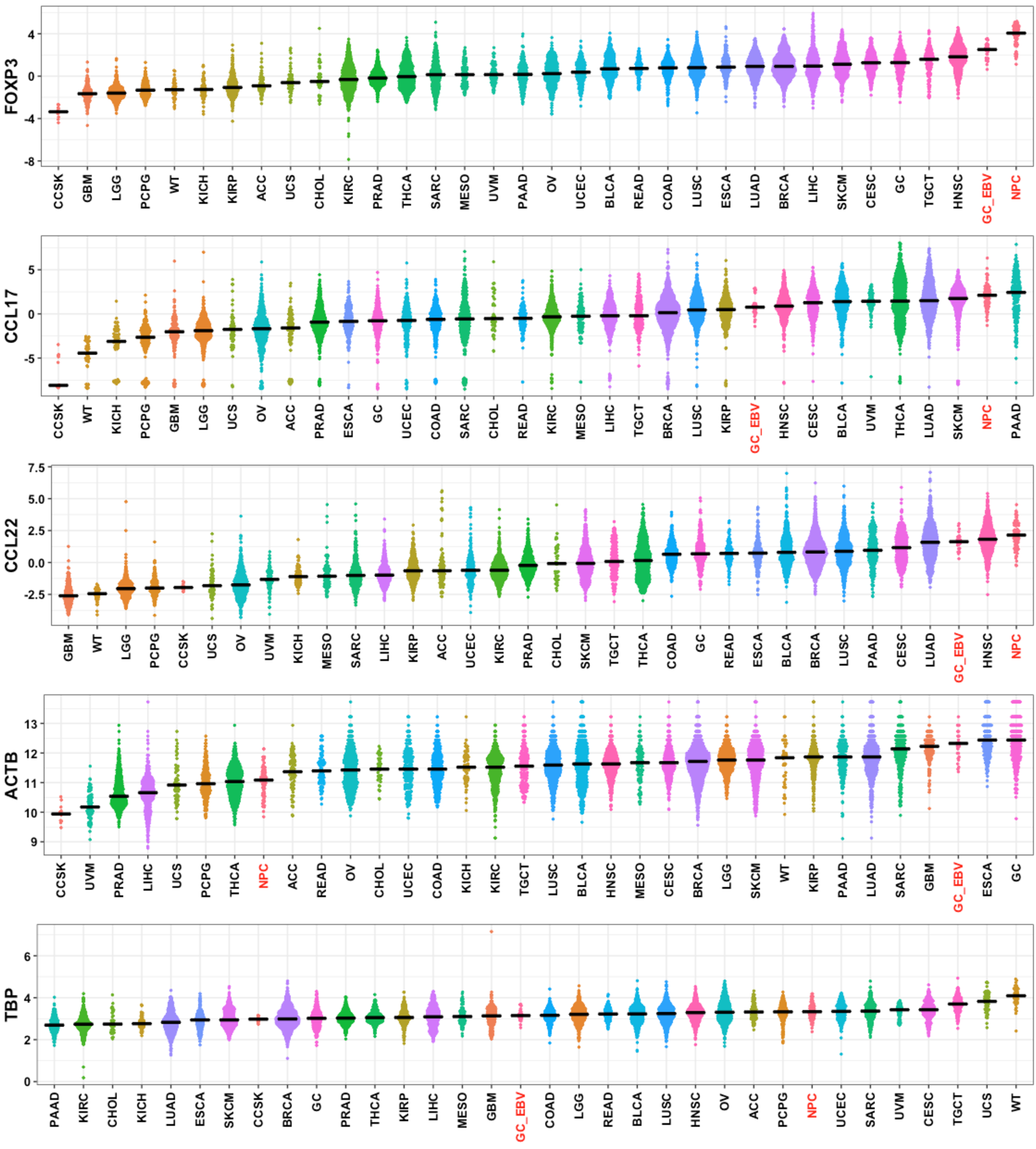

Supplement: S3 Fig — Data is shown as in Fig 3, with the addition of control “housekeeping” genes β-Actin (ACTB) and TATA-Box Binding Protein (TBP). Tumor types are sorted by increasing median expression and plotted as log2 Transcripts per Million for each gene. Tumor abbreviations are defined in S1 Table. (TIF) [file ppat.1010200.s003.tif]

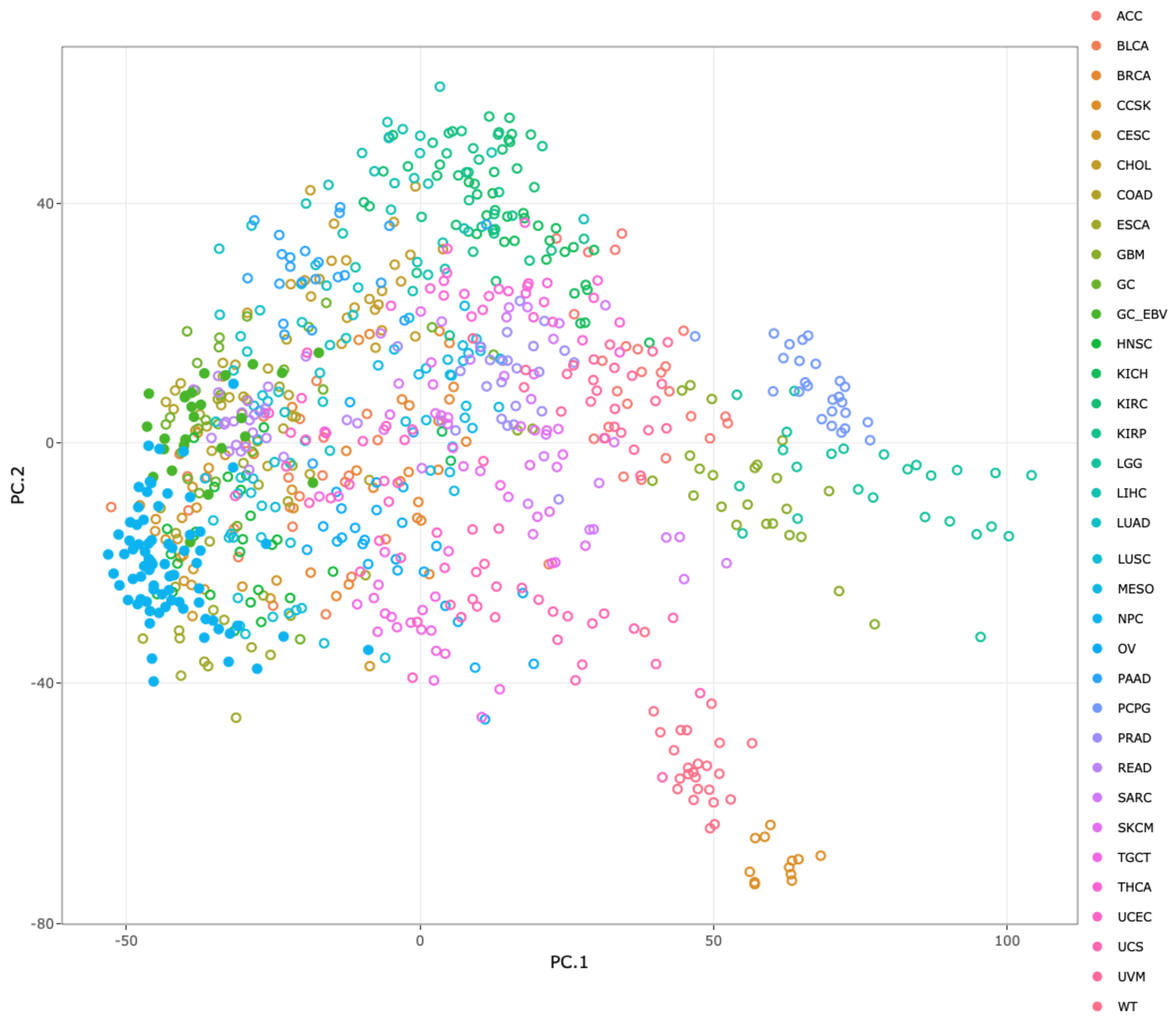

Supplement: S4 Fig — Principal Component Analysis was performed on the tumor expression data from which Fig 3A and S3 Fig are derived, with the first two principal components (PC.1 and PC.2) plotted along the X and Y axes, respectively. EBV+ tumor types are shown in filled circles, all others in open circles. The NPC and GC_EBV samples are distributed amongst other tumor types, and in close proximity to EBV- Gastric (GC) and Head & Neck (HNSC) carcinomas. Tumor abbreviations are defined in S1 Table. (TIF) [file ppat.1010200.s004.tif]

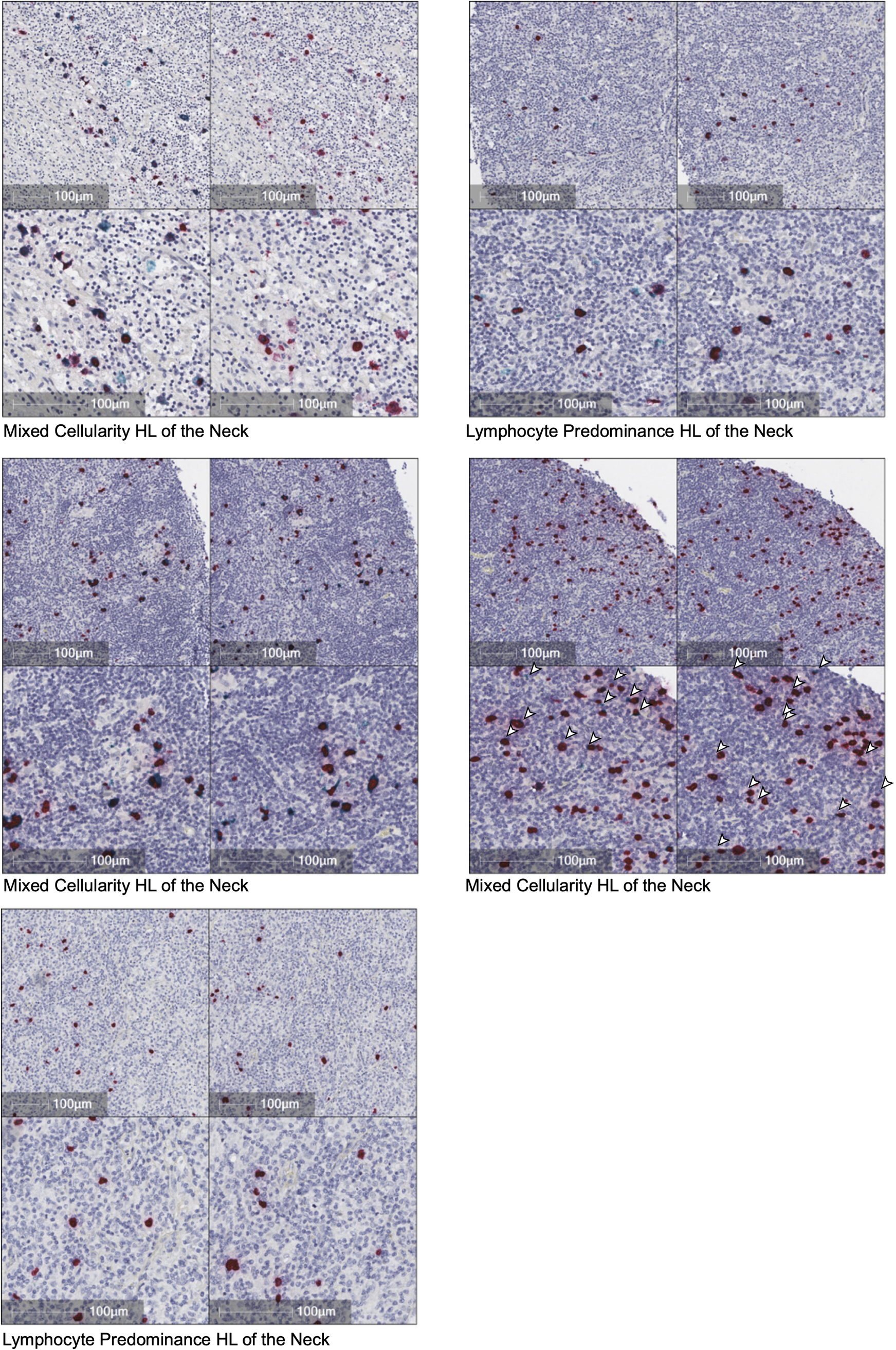

Supplement: S5 Fig — Top left: 20x magnification of staining for EBER1 (red) and CCL22 (cyan); top right: 20x EBER1 (red) and CCL17 (cyan); bottom left and right: 40x magnification of the upper images. Nuclear haematoxylin staining is in blue. Arrows highlight CCL17 and CCL22 in the fourth biopsy sample. CCL17 and CCL22 signals can be seen in all but the 5th biopsy sample. (TIF) [file ppat.1010200.s005.tif]

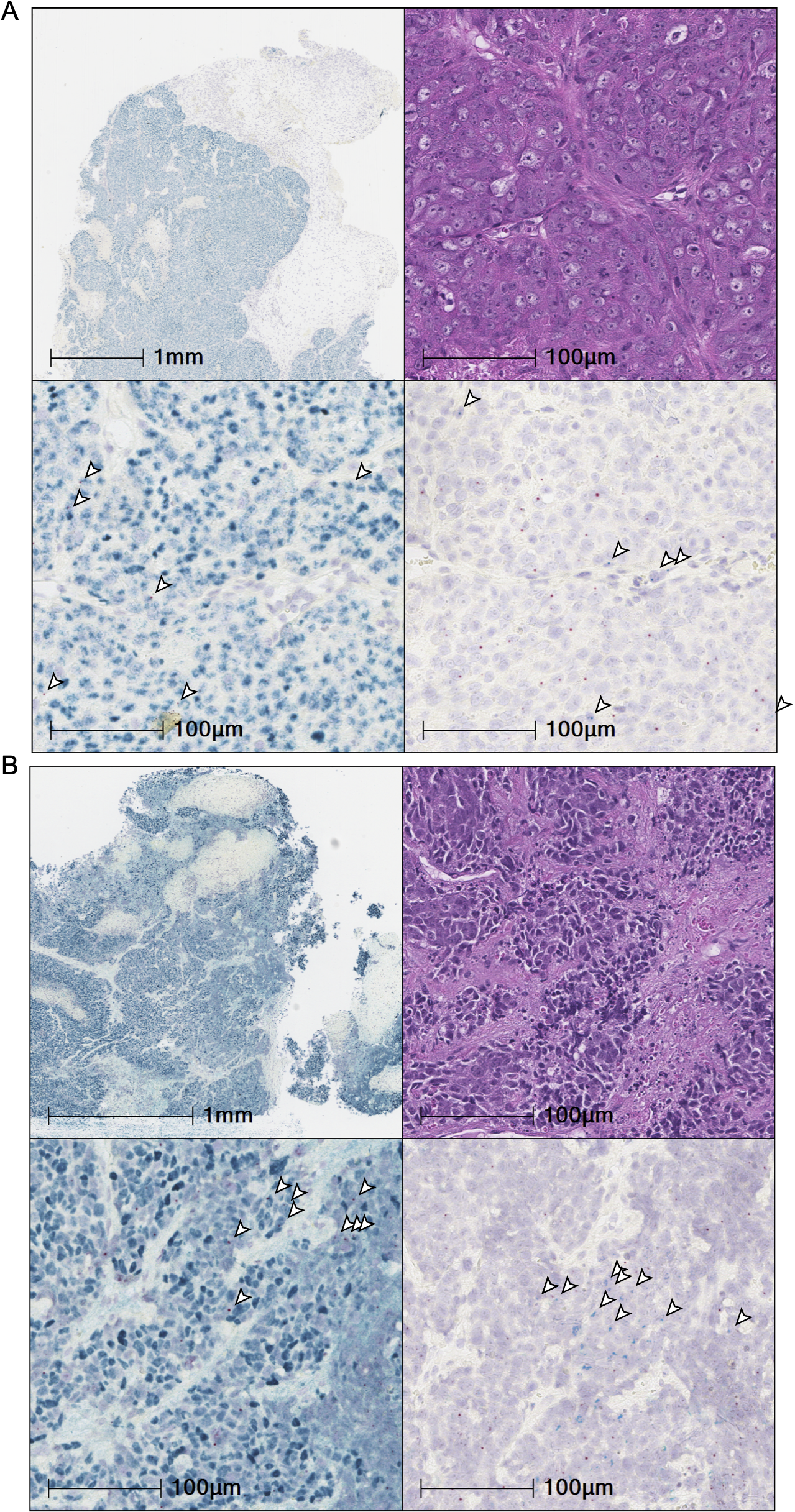

Supplement: S6 Fig — Representative images of duplex RNA in situ hybridization of a (A) C17 NPC xenograft and a (B) C18 NPC xenograft are shown. Images shown are of corresponding serial sections: H&E (top right); EBER1 (cyan) and human CCL22 (red) (top and bottom left); and mouse CCL22 (cyan) and human CCL22 (red) (bottom right). Arrows highlight select human CCL22 (bottom left panels) and mouse CCL22 (bottom right panels) staining. Staining in bottom panels is digitally enhanced (unenhanced versions in S8A and S8B Fig). Nuclear haematoxylin staining appears in pale blue. (TIF) [file ppat.1010200.s006.tif]

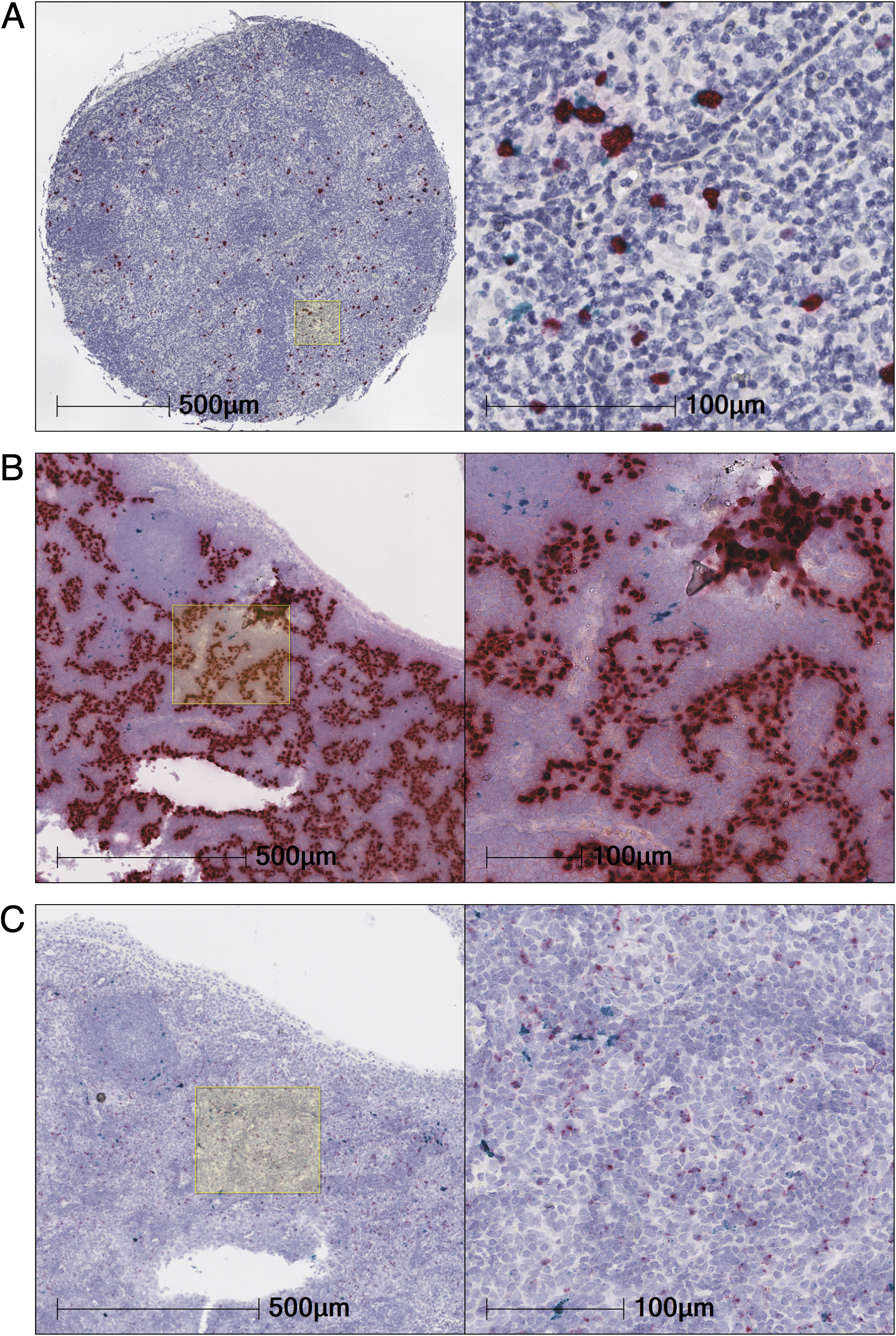

Supplement: S7 Fig — Representative images of RNA in situ hybridization (ISH) of (A) Hodgkin lymphoma (HL) and (B) nasopharyngeal carcinoma (NPC) samples probed for EBER1 (red) and CCL22 (cyan) are shown. (C) A matched section serial to that in (B) was probed for FOXP3 (red) and CCL22 (cyan). Yellow boxes in lower magnification views (left) indicate sources of magnified regions shown on right. Nuclear haematoxylin staining is shown in pale blue. These unprocessed images exported by HALO software correspond to the color-enhanced versions shown in Fig 4. (TIFF) [file ppat.1010200.s007.tiff]

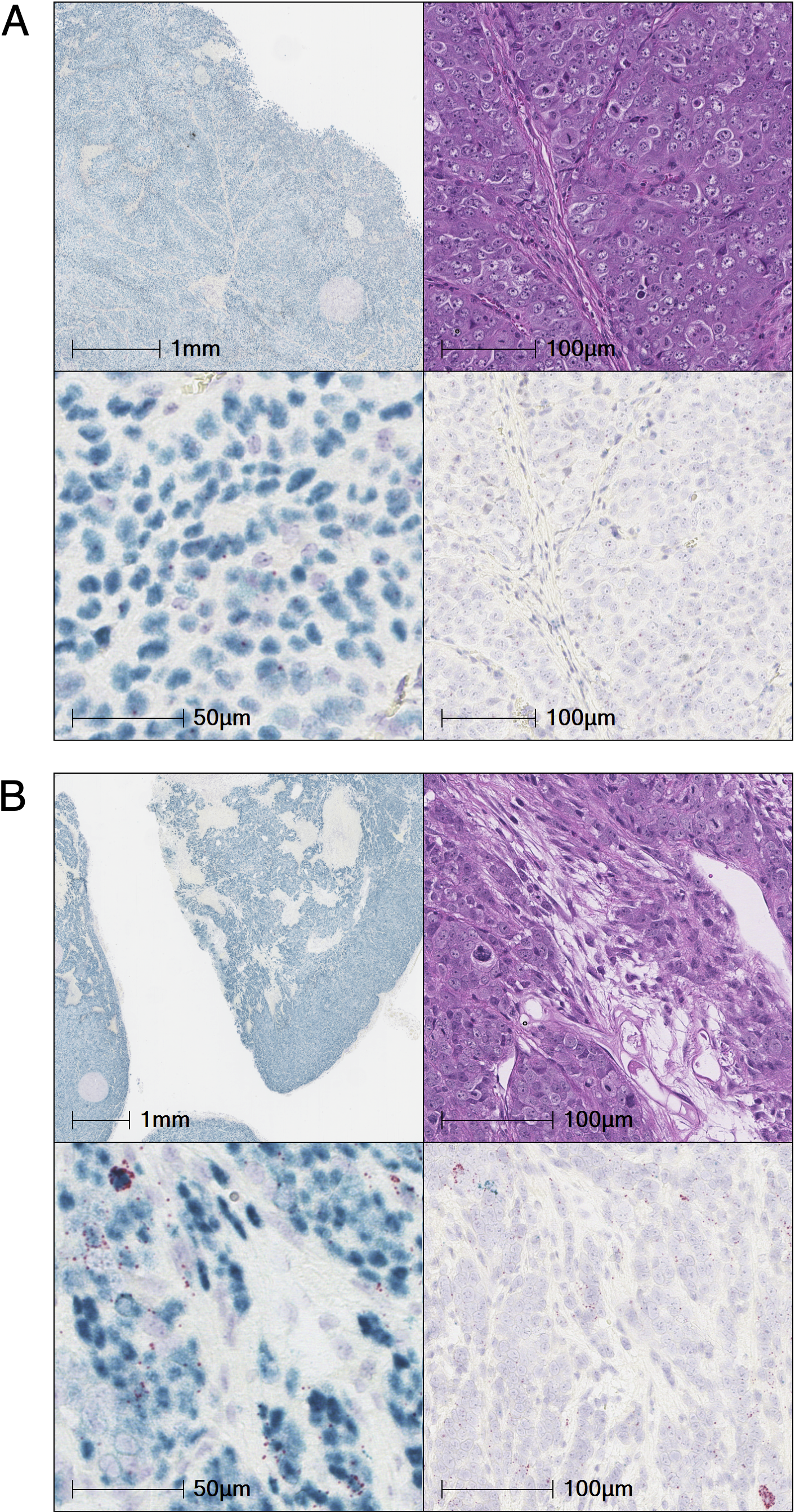

Supplement: S8 Fig — Representative images of RNA in situ hybridization (ISH) of a (A) C15 NPC xenograft and a (B) C666-1 NPC xenograft are shown. Images shown are of corresponding serial sections: H&E (top right); EBER1 (cyan) and human CCL22 (red) (top and bottom left at two magnifications; and mouse CCL22 (cyan) and human CCL22 (red) (bottom right). Nuclear haematoxylin staining shown in pale blue. These unprocessed images exported by HALO software correspond to the color-enhanced versions shown in Fig 5. (TIFF) [file ppat.1010200.s008.tiff]

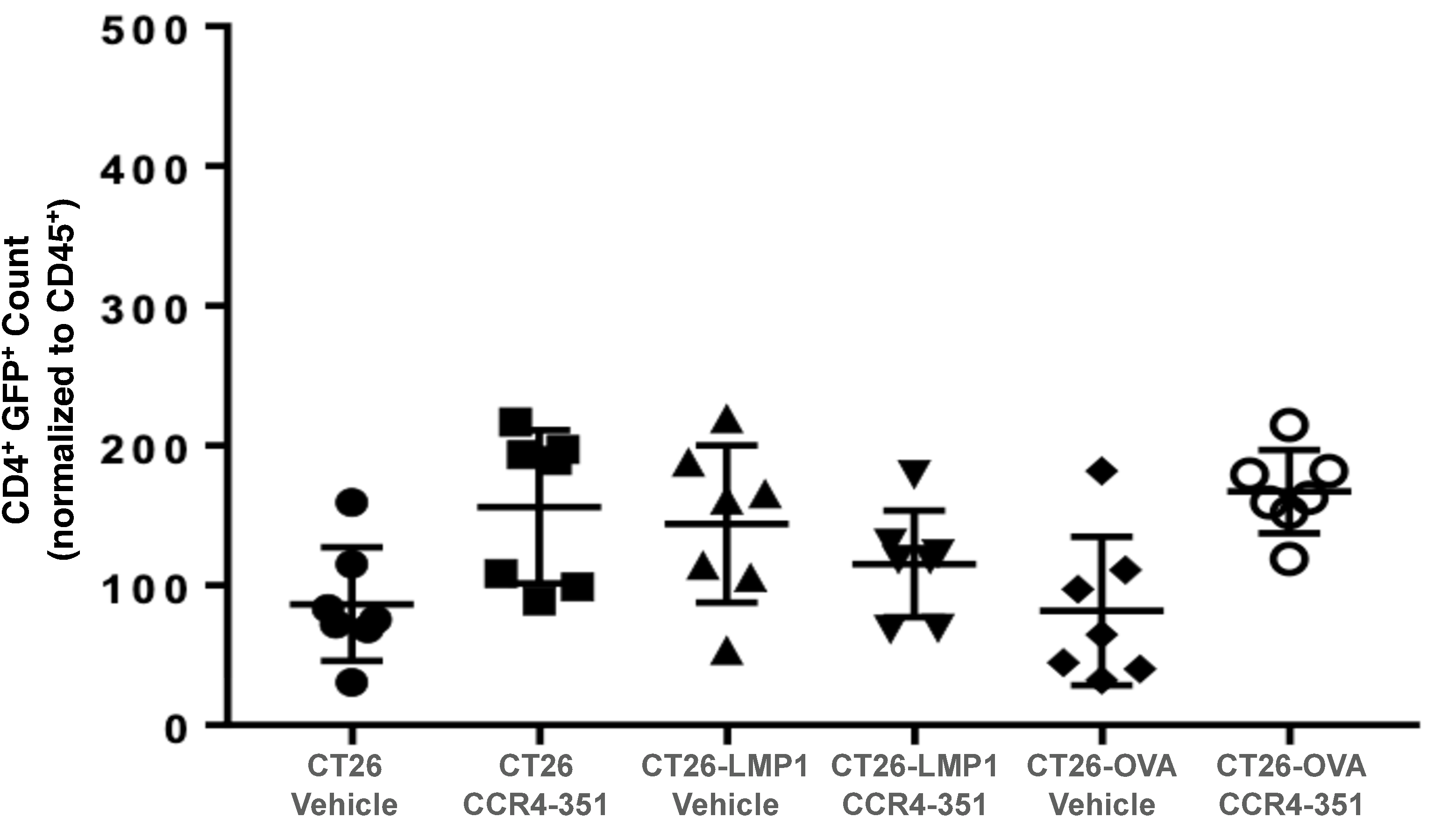

Supplement: S9 Fig — The numbers of iTreg from spleen, identified as CD4+ GFP+ cells, normalized to total CD45+ cell count, were quantified by FACS analysis. (TIF) [file ppat.1010200.s009.tif]

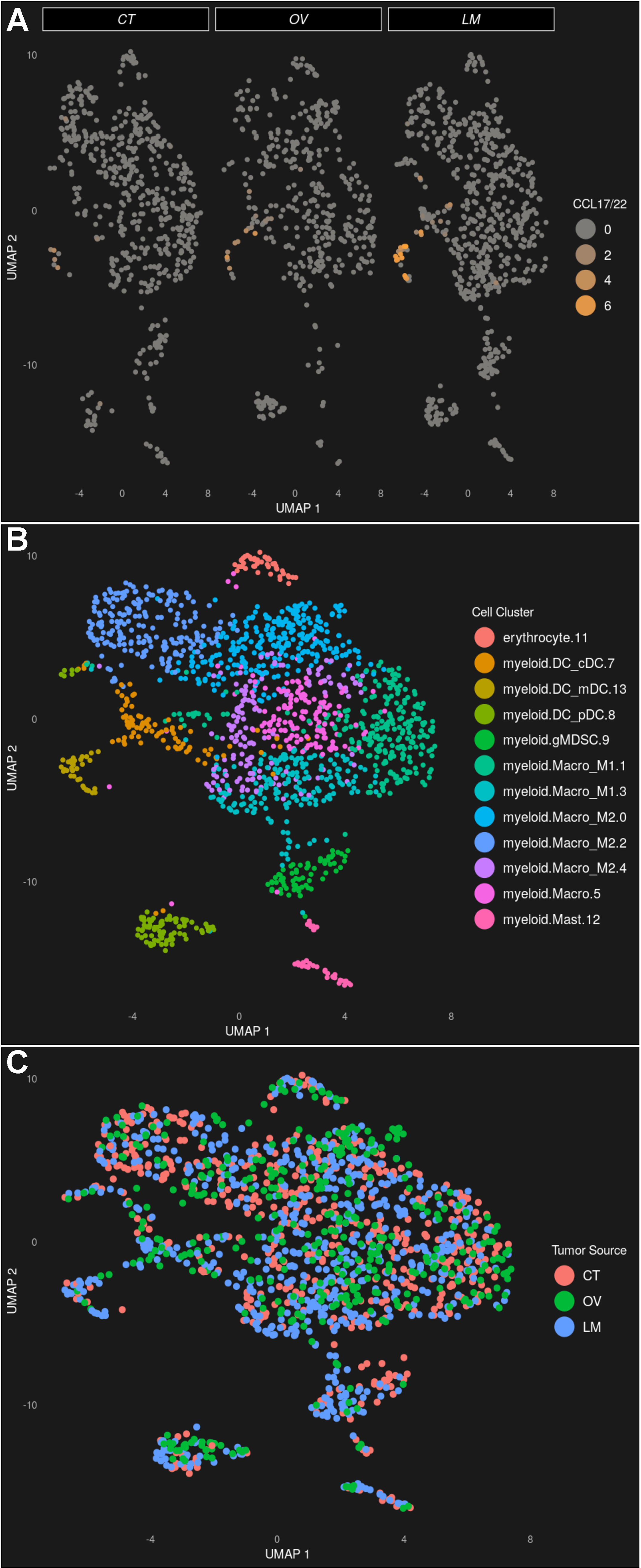

Supplement: S10 Fig — The UMAP 2D projection of all myeloid-compartment cells is colored by combined CCL17 and CCL22 expression (TPM) and faceted by tumor (A), colored by cell cluster (B), or colored by tumor source (C). CT = CT26, OV = CT26-OVA, LM = CT26-LMP1 (TIF) [file ppat.1010200.s010.tif]

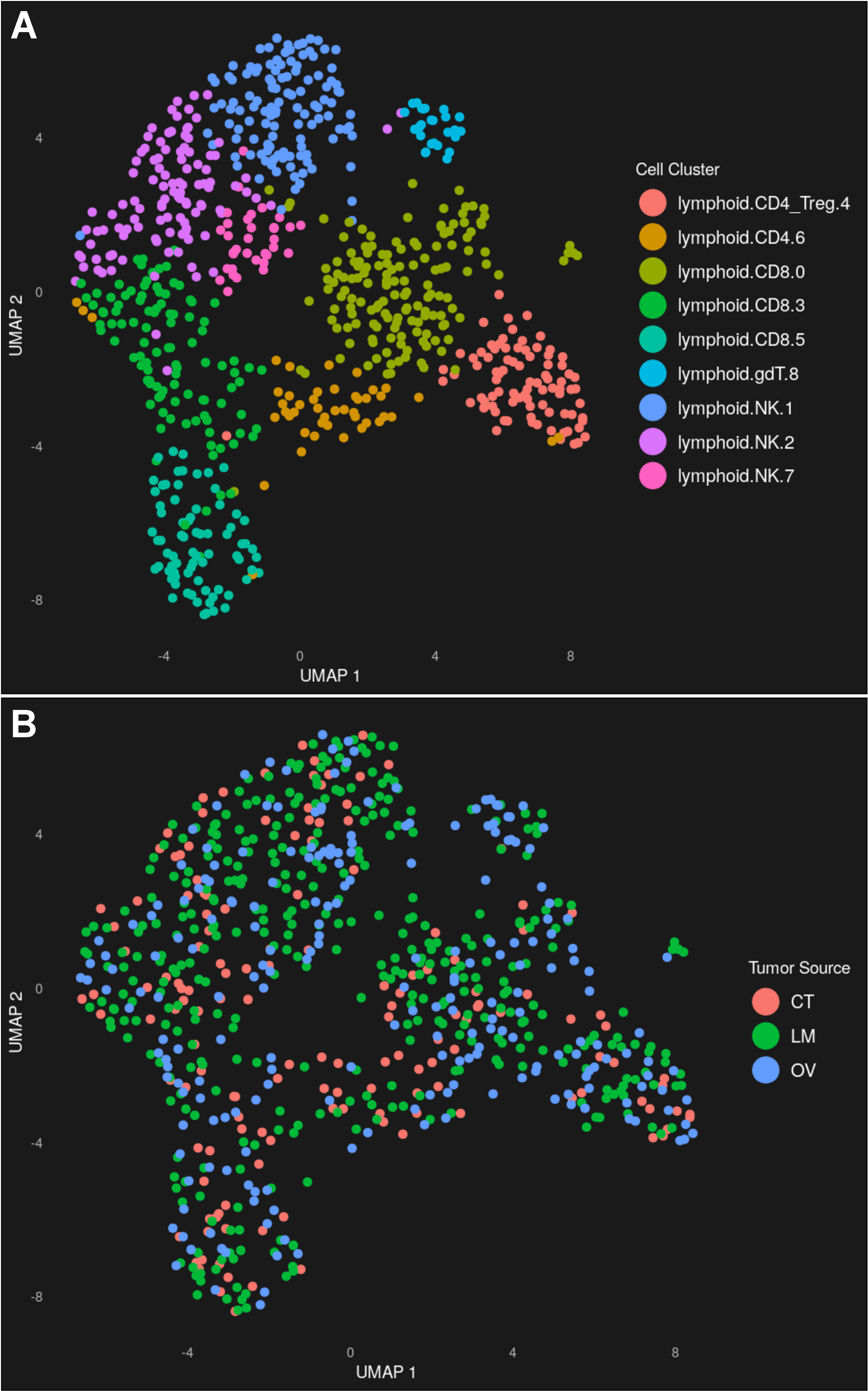

Supplement: S11 Fig — The UMAP 2D projection of all lymphoid-compartment cells is colored by cell cluster. The lymphoid UMAP projection is colored by cell cluster (A) or by tumor source (B). CT = CT26, OV = CT26-OVA, LM = CT26-LMP1 (TIF) [file ppat.1010200.s011.tif]

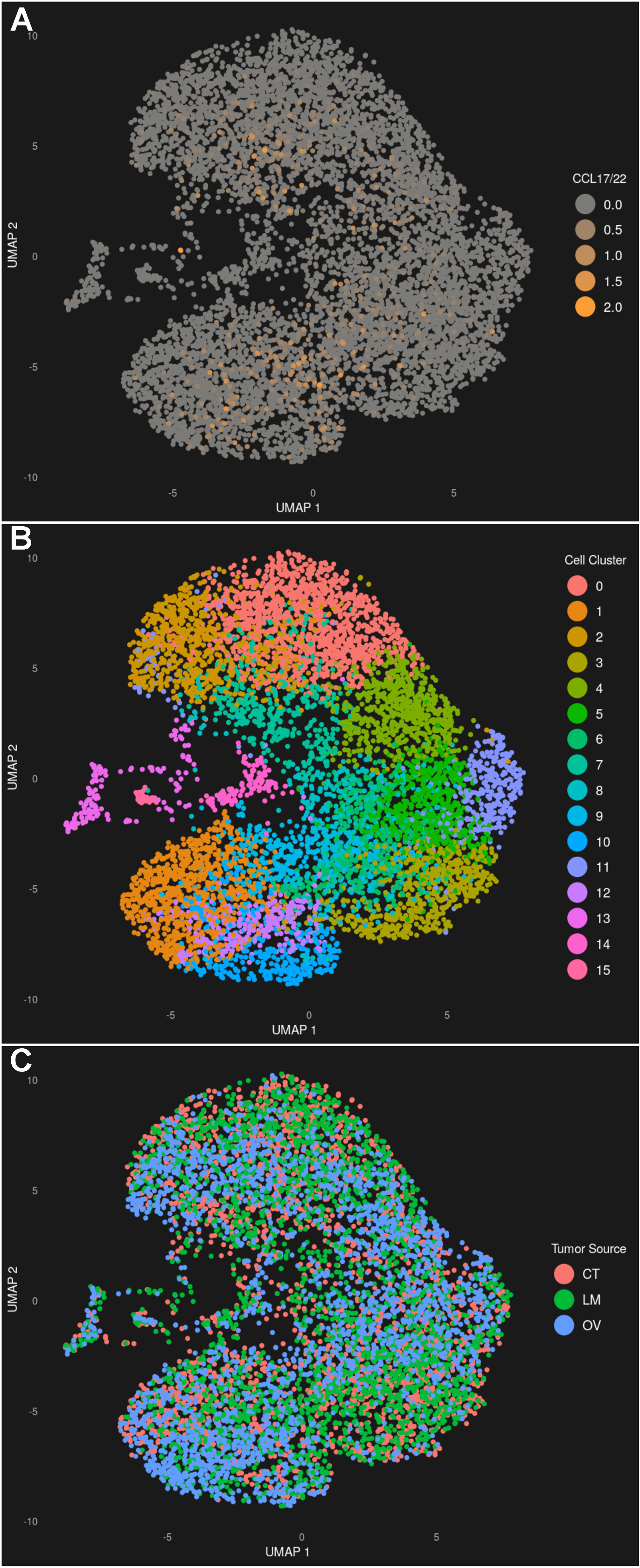

Supplement: S12 Fig — The UMAP 2D projection of all myeloid-compartment cells is colored by combined CCL17 and CCL22 expression (TPM) (A), colored by cell cluster (B), or colored by tumor source (C). CT = CT26, OV = CT26-OVA, LM = CT26-LMP1 (TIF) [file ppat.1010200.s012.tif]

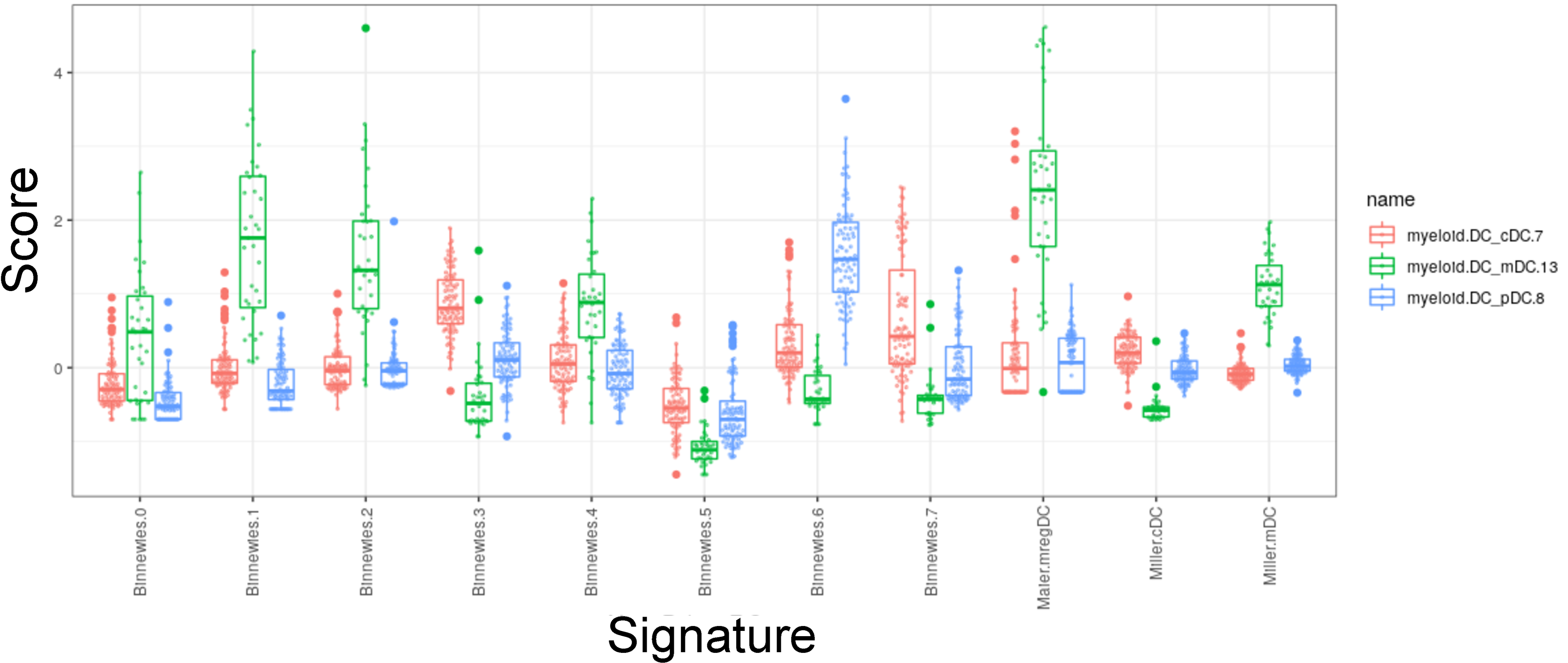

Supplement: S13 Fig — Gene signatures from Binneweis et al, Maier et al, and Miller et al (S4 Table) were applied to the three DC-like clusters identified in this study. A robust Z-transform was used on the relevant genes across all myeloid cells, then the average Z scores for the genes in each signature were plotted for myeloid cluster 1 (migratory DC, mDC), myeloid cluster 7 (classical resident DC, cDC), and myeloid cluster 8 (plasmacytoid DC, pDC). The cDC cluster stood out for Binnewies signature 3 (resident CD11b+ cDC2), Binnewies signature 7 (resident CD8a+ cDC1), and the Miller cDC signature. The mDC cluster stood out for Binnewies signature 1 (migratory CD103+ cDC1), Binnewies signature 2 (Langerhans cell), Miller mDC, and the Maier mregDC signature. The pDC assignment is supported by expression of Tlr7 and Tlr9 in this cell cluster. (TIF) [file ppat.1010200.s013.tif]

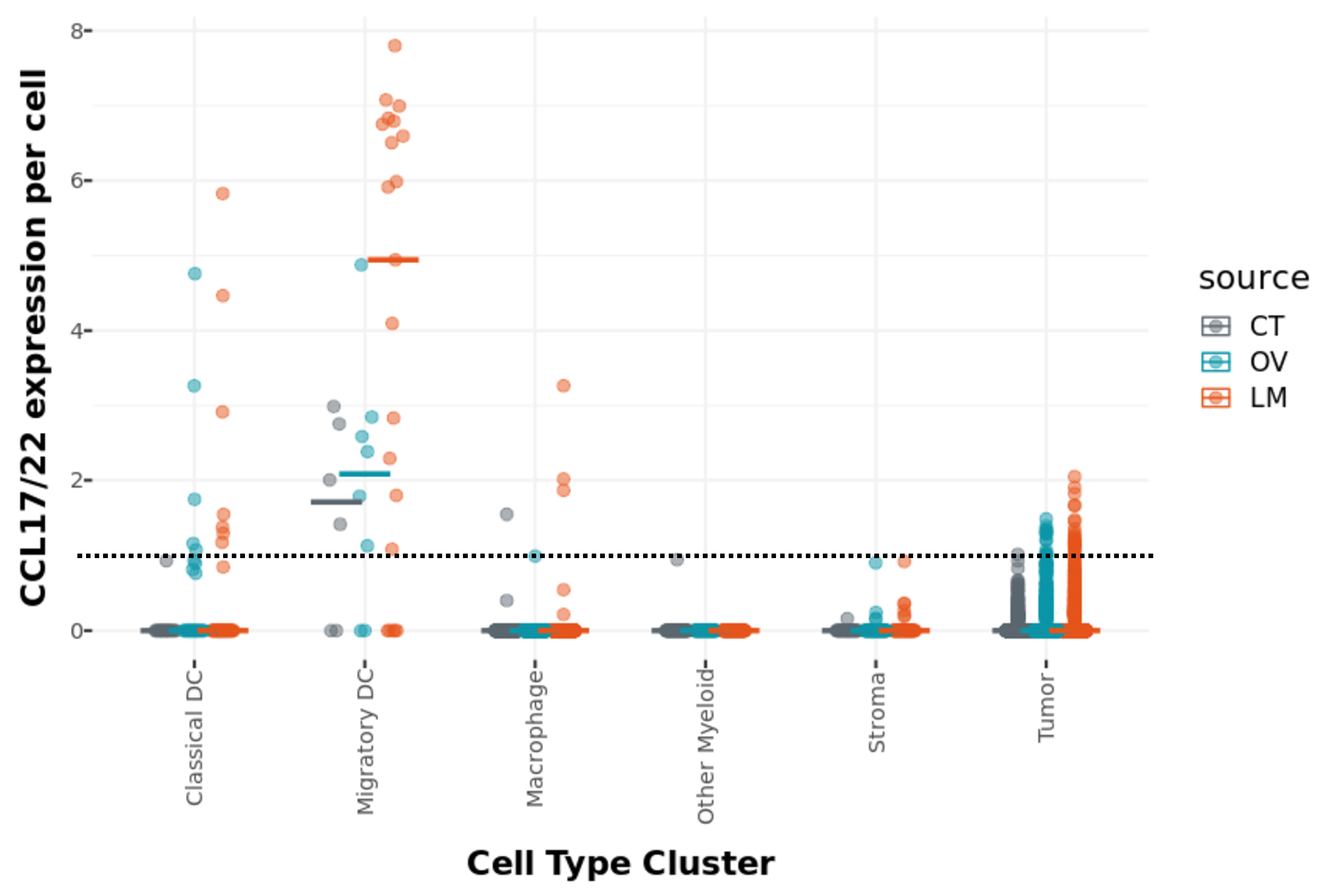

Supplement: S14 Fig — Combined CCL17 and CCL22 expression for each CCL17/22-expressing cell from each tumor sample are plotted as Transcripts per Million (TPM). Short horizontal lines indicate median expression values per population. A TPM of 1.0 (dashed line) was arbitrarily considered to be "productive" expression for further filtering. CT = CT26, OV = CT26-OVA, LM = CT26-LMP1 (TIF) [file ppat.1010200.s014.tif]
