# Supplementary material for: EBV+ tumors exploit tumor cell-intrinsic and -extrinsic mechanisms to produce regulatory T cell-recruiting chemokines CCL17 and CCL22
Source: PLoS Pathog. 2022 Jan 13;18(1):e1010200. doi: 10.1371/journal.ppat.1010200 (PMC8791514; doi:10.1371/journal.ppat.1010200)
Supplement: Attachment — Submitted filename: PLOS Pathogens reviewer comments finalized.docx [file ppat.1010200.s019.docx]

We thank the Reviewers for their time and diligence in reviewing our manuscript. The Reviewers’ comments are reproduced below in blue, interspersed with our responses in black.

**Part II – Major Issues: Key Experiments Required for Acceptance**
Please use this section to detail the key new experiments or modifications of existing experiments that should be absolutely required to validate study conclusions.

Reviewer #1: 1. sFig1 and lane 115 to 117) In contrast to previous reports the gastric carcinoma line NCI-N87 is now found to be EBV+. Are NCI-N87 cells used in this work derived from a certified source? This cell line should be verified.

As described in the manuscript’s Materials and Methods, this cell line was ordered directly from ATCC, frozen at 3-5 passages, and used at passage 4-6. ATCC authenticates all of their cell lines and we generally rely on their quality control practices to certify that cell line identifications are correct. Due to questions about this cell line, we did follow up STR profiling using the services of IDEXX that further confirmed the identity of our sample of NCI-N87.

2. Fig 1C) The mechanism of chemokine induction by LMP1/LMP2A is not sufficiently adressed. LMP2A seems to considerably contribute to CCL17 expression in Raji. Jijoye and NC-37 express low LMP2A levels. Is LMP1 sufficient to induce chemokines in these cells or may other mechanisms contribute? The authors should express LMP1, LMP2A and a combination of both in EBV-negative cells such as Ramos or NPC cells and analyse CCL17 levels. Also LMP1 with mutations in CTAR1 and/or CTAR2 could then be easily tested.

We agree with the reviewer that our manuscript did not go into a detailed mechanistic analysis of CCL17/CCL22 induction by LMP1/LMP2A and that such an analysis would be very interesting. However, our goal with this work was to dissect the immunological implications, both in terms of immune evasion and potential pharmacological abrogation thereof, of EBV+ tumor phenotypes. The more superficial mechanistic work we did present on LMP1/LMP2A was primarily aimed at allowing us to understand which EBV+ tumors were most likely to be expressing CCR4 ligands and guided us to generating the CT26-LMP1 tumor model. We hope that the reviewer will accept that this deep mechanistic study of LMP1 and LMP2 biology is tangential to the core aims of this work and is best reserved for a follow-up publication.

Reviewer #2: 1. It is not clear to this reviewer if the authors are stating that TREG tumour infiltration was induced or due to migration. If this cannot be inferred, please clarify this in the discussion.

We believe that the close coupling of the migration-inducing chemokines CCL17 and CCL22 with Treg infiltrate and lack of such a correlation with TGFβ expression suggest that migration is the major driver of Treg numbers in tumors.

With reference to this study, a simple Student’s t-test looking for differences in the expression of Tgfb1 between the infiltrating cells in the CT26 and CT26-LMP1 samples, as measured by single cell RNA-sequencing, finds nothing significant. Thus, at least in this model, our data suggest migration (due to the significant increases in CCR4 ligands) and not induction. Those results are shown here:

| cell_cluster | p_val | fdr |
| --- | --- | --- |
| myeloid.Macro_M2.0 | 0.023 | 0.51 |
| lymphoid.CD8.3 | 0.055 | 0.52 |
| myeloid.Macro.5 | 0.071 | 0.52 |
| myeloid.Macro_M1.1 | 0.21 | 0.86 |
| lymphoid.CD8.5 | 0.24 | 0.86 |
| lymphoid.CD4_Treg.4 | 0.26 | 0.86 |
| myeloid.DC_pDC.8 | 0.32 | 0.86 |
| lymphoid.NK.2 | 0.32 | 0.86 |
| lymphoid.CD4.6 | 0.41 | 0.86 |
| myeloid.Macro_M1.3 | 0.46 | 0.86 |
| lymphoid.CD8.0 | 0.48 | 0.86 |
| myeloid.Macro_M2.2 | 0.50 | 0.86 |
| tumor | 0.52 | 0.86 |
| myeloid.Mast.12 | 0.55 | 0.86 |
| lymphoid.NK.1 | 0.65 | 0.92 |
| stroma.Fibroblast.6 | 0.74 | 0.92 |
| myeloid.gMDSC.9 | 0.78 | 0.92 |
| stroma.Endothelial.10 | 0.82 | 0.92 |
| lymphoid.NK.7 | 0.83 | 0.92 |
| myeloid.Macro_M2.4 | 0.87 | 0.92 |
| myeloid.DC_mDC.13 | 0.88 | 0.92 |
| myeloid.DC_cDC.7 | 0.98 | 0.98 |

Looking into the tumor cell expression component of the single cell RNA-Seq, the DESeq2 algorithm also shows no significant difference between Tgfb1 expression in CT26 and CT26-LMP1 cells. An increase is seen in CT26-LMP1 vs CT26-OVA, but this narrowly misses statistical significance and wouldn’t support the hypothesis of TGFβ explaining increased Treg numbers anyway.

| comparison | p_val | avg_logFC | p_val_adj |
| --- | --- | --- | --- |
| CT26 v CT26-LMP1 | 0.67 | 0.21 | 1 |
| CT26 v CT26-OVA | 0.36 | 0.27 | 1 |
| CT26-LMP1 v CT26-OVA | 0.0000028 | 0.064 | 0.0895 |

With respect to other data sets, we have previously analyzed the 10,000+ tumors in The Cancer Genome Atlas (TCGA), and found strong correlations between Treg (as estimated by FOXP3 expression) and CCL17 and CCL22, but not with TGFB1 expression (Marshall, L. A. *et al.* Tumors establish resistance to immunotherapy by regulating Treg recruitment via CCR4. *J Immunother Cancer* **8**, e000764; 2020)).

A comparison of TCR repertoire diversity in human breast cancers to circulating Treg also supports the idea that the tumor-Treg burden is largely migration-driven (Plitas, G. *et al.* Regulatory T Cells Exhibit Distinct Features in Human Breast Cancer. *Immunity* **45**, 1122–1134; 2016)).

Text to this effect has been added to the Discussion.

2. Please discuss the mechanisms by which EBV might influence tumour extrinsic chemokine production.

Our data support a model of increased numbers of and more suppressive phenotypes for migratory Dendritic Cells in the tumor microenvironment of EBV+ tumors and that this effect can be driven by LMP1 expression. Our data demonstrate that this occurs in a mouse tumor model, and this effect recapitulates aspects of the EBV+ tumor microenvironment (TME) observed in human tumors. The Ovalbumin antigen control in our mouse model suggests that this observed effect is not simply due to the presence of a foreign antigen in the TME, but extensive further experimentation will be required to further narrow down the mechanism(s). A variety of ligands have been identified that stimulate the migration of DCs, including chemokines such as CCL19 and CCL21 and small molecules such as Leukotriene B4 and oxysterols (via the intriguingly-named Epstein Barr Virus Induced Gene 2 (EBI2, aka GPR183); Randolph, G. J., Ochando, J. & Partida-Sánchez, S. Migration of Dendritic Cell Subsets and their Precursors. *Annu Rev Immunol* **26**, 293–316 (2008); Feng, M. *et al.* Regulation of the Migration of Distinct Dendritic Cell Subsets. *Frontiers Cell Dev Biology* **9**, 635221 (2021)) . We did not observe increased expression of candidate signaling proteins in our data, although a sequencing study with larger cell numbers, a candidate gene expression analysis approach, a protein detection methodology, and mass spectrometry analysis for non-protein ligands would be required to more definitively detect or rule out changes in these analytes. We welcome follow-up experimental work that can help to elucidate the relevant mechanisms.

We have updated the Discussion with this information.

3. Figure 4 and 5 are difficult to understand and view. Images (particularly on the right hand) are blurred, and colours are indistinct from each other, and the legends do not explain what we are meant to be observing. This needs considerable attention and re-working.

Unfortunately, the nature of chromogenic *in situ* hybridization, particularly for FFPE slides, is that colors overlap and cell and nuclei boundaries are not visually crisp. In this case, since EBER1 expression is so much stronger than that of the other analytes, visualization of overlapping stains is especially challenging. We had performed image post-processing to enhance the fainter-staining probes to an amount that we felt would not misrepresent the true data while also providing the original data in the Supplementary Figures.

As there are different types of panels on the right hand side, we are not sure exactly which the Reviewer finds issues with. However, we fully understand the general issue that the Reviewer raises. Our best way to address this is to remove the lower magnification and H&E sections from the images, allowing us the real estate to enlarge the key panels (right panels of Figures 4A, 4B, and 4C and bottom panels of Figures 5A and 5B). We also further color-enhanced these scans. The original, unprocessed images and the full complement of panels are still supplied in Supplementary Figures 7 and 8. We hope that these changes will make viewing and interpreting the images easier for the reader.

We do observe that the merged pdf file provided by PLOS contains a blurrier, apparently lower-resolution, version of the images that we submitted. Please use the links to download the tiff files as submitted by us to see the images as they are intended to be viewed.

**Part III – Minor Issues: Editorial and Data Presentation Modifications**
Please use this section for editorial suggestions as well as relatively minor modifications of existing data that would enhance clarity.

Reviewer #1: 1. Statistics: Which T-Test was used?

All t-tests were standard two-sample Student’s t-tests. The Statistics section of the Materials and Methods has been updated to state this.

2. 1A) Technical or biological replicates? Where do the LMP1/LMP2A expression data come from? Statistics is required.

All replicates are biological replicates – we have clarified this in the manuscript. As specified in the Figure 1 legend: “Western blots on 50 μg of protein lysate from 9 human cell lines were probed for LMP1 and LMP2A [and] quantitated by densitometry.“ It is unclear what statistics the Reviewer is interested in, we do not make any statistical claims from this data, merely qualitative observations. If there is a specific statistical test that the Reviewer feels is appropriate, we would be happy to perform it.

3. sFig1A) Are all panels shown for a specific antibody derived from the same blot? Are the protein levels comparable?

All panels per antibody are from the same blot for the first 7 lanes and from a separate blot for the last 2 lanes. This is indicated by the black borders around the two sets. The Reviewer raises a very valid point regarding the construction of the figure and we have clarified this in the figure legend. The lanes have been rearranged (as indicated by gaps) in order to align them across the different antibody rows – LMP1 and HSP90 were probed on one blot and LMP2A, from the same lysate source, on a second blot. The protein levels are comparable, as can be visualized by similar staining of the HSP90 loading control.

4. Fig 1B) Does control LMP2A siRNA affect LMP1 expression?

Yes, the control LMP2A siRNA does affect LMP1 expression. We make note of this in the manuscript. siRNA almost always give some level of off-target effect, unfortunately in this case the off-target was a gene of interest. This off-target LMP1 reduction is to a lesser degree than the on-target LMP1 siRNA effect and does not have a biological effect on our assays (CCL17 and CCL22 protein production).

5. Fig 4) What is known about the status of LMP1/LMP2A expression in the HL and NPC biopsies?

EBER1 was assayed in these experiments to understand the overall levels of EBV transcript, but LMP1 and LMP2A were not assayed in our hands. However, from the review by Lo *et al.* (The Role of EBV-Encoded LMP1 in the NPC Tumor Microenvironment: From Function to Therapy. *Frontiers Oncol* **11**, 640207; 2021), it has been reported that LMP1 is detected in nearly all premalignant and preinvasive NPC tissue samples but in 20% to 60% of NPC cases. Interestingly, LMP1 expression is associated with a poor survival prognosis in NPC.

In the case of HL, in a study by Hasmi *et al.* (Latent membrane protein 1 (LMP1) expression in Hodgkin lymphoma and its correlation with clinical and histologic parameters. *World J Surg Oncol* **15**, 89; 2017) LMP1 expression was observed in 45 out of 66 cases (68%). In this work, no association of LMP1 expression with survival or other clinical parameters was observed.

6. Fig 8) Statistics is required.

It is unclear what statistics the Reviewer is interested in seeing. We make no statistical claims with regards to the data shown in Figure 8, simply a qualitative observation that a dose-dependent increase in chemokine secretion with IFNγ stimulation is seen in all cell lines. We are happy to calculate any desired statistics upon further clarification by the Reviewer.

Reviewer #2: Was CCL17 proportional to the density of Raji cells in culture (as per CCL22).

Supplementary Figure 1C shows a proportional increase in CCL22 production with increased numbers of Raji cells. We did not assay CCL17 in this experiment, though we would expect to see a similar pattern.

Legend to Fig 2: explain CCRE CEM cells as CD4 lymphoblasts.

The manuscript has been updated to include this information.
