# Supplementary material for: EBV+ tumors exploit tumor cell-intrinsic and -extrinsic mechanisms to produce regulatory T cell-recruiting chemokines CCL17 and CCL22
Source: PLoS Pathog. 2022 Jan 13;18(1):e1010200. doi: 10.1371/journal.ppat.1010200 (PMC8791514; doi:10.1371/journal.ppat.1010200)
Supplement: Attachment — Submitted filename: Reply to Reviewer Comments.docx [file ppat.1010200.s020.docx]

Dear Editors and Reviewers,

Please see our comments below, in red, regarding the latest revision of the manuscript to address Reviewers’ comments.

Reviewer #1: 1. Statistics of Figure 1A): **Significance of the claimed major differences should be verified by T-Tests and p-values should be given.**

Our claim regarding Figure 1A was of “a very close match between LMP1 expression and both CCL17 and CCL22.” We felt that a correlation analysis was a better statistical test to assess the match than a t-test. Therefore, we calculated Pearson correlations between these parameters and have reported these in the manuscript:

Pearson correlations of 0.96 (p value = 2.3e-6) and 0.95 (p value = 3.4e-5) were found between CCL17 and LMP1, and CCL22 and LMP1, respectively. No correlations with LMP2A were significant (p values > 0.1).

Additional detail on this statistical analysis is reported in the Statistics section of the Materials & Methods

Significance for LMP proteins and chemokines reported for Fig 1A was calculated by the R “stats::cor.test” function using Pearson correlations and two-sided hypothesis testing.

2. Statistics of Figure 8: **Standard deviations should be shown. Is there a significant difference between CT26, OVA and LMP1? From this reviewer's point of view, this could be tested by ANOVA comparing the LMP1 with the CT26, OVA curves, or by T-Tests at a distinct IFNg concentration**. In case of doubt, a statistician should be consulted.

In order to properly satisfy the Reviewer’s desire for statistical analysis of the CCL17 expression curves, we sought to go back to the raw ELISA signal data rather than the calculated CCL17 concentrations. However, inspection of the raw data made us question its quality and out of an abundance of caution we decided to repeat the entire experiment. Despite several attempts, the experiment we could not repeat the induction of CCL17 expression in CT26 cells by IFNγ. Therefore we are dropping Figure 8 from the manuscript and any discussion thereof.

Figure Files:

While revising your submission, please upload your figure files to the Preflight Analysis and Conversion Engine (PACE) digital diagnostic tool, <https://pacev2.apexcovantage.com>. PACE helps ensure that figures meet PLOS requirements. To use PACE, you must first register as a user. Then, login and navigate to the UPLOAD tab, where you will find detailed instructions on how to use the tool. If you encounter any issues or have any questions when using PACE, please email us at [figures@plos.org](mailto:figures@plos.org).

Data Requirements:

Please note that, as a condition of publication, PLOS' data policy requires that you make available all data used to draw the conclusions outlined in your manuscript. Data must be deposited in an appropriate repository, included within the body of the manuscript, or uploaded as supporting information. This includes all numerical values that were used to generate graphs, histograms etc.. For an example see here: <http://www.plosbiology.org/article/info%3Adoi%2F10.1371%2Fjournal.pbio.1001908#s5>.

Reproducibility:

To enhance the reproducibility of your results, we recommend that you deposit your laboratory protocols in [protocols.io](http://protocols.io), where a protocol can be assigned its own identifier (DOI) such that it can be cited independently in the future. Additionally, PLOS ONE offers an option to publish peer-reviewed clinical study protocols. Read more information on sharing protocols at<https://plos.org/protocols?utm_medium=editorial-email&utm_source=authorletters&utm_campaign=protocols>

References:

Please review your reference list to ensure that it is complete and correct. If you have cited papers that have been retracted, please include the rationale for doing so in the manuscript text, or remove these references and replace them with relevant current references. Any changes to the reference list should be mentioned in the rebuttal letter that accompanies your revised manuscript. If you need to cite a retracted article, indicate the article’s retracted status in the References list and also include a citation and full reference for the retraction notice.
